# “When” Does Picture Naming Take Longer Than Word Reading?

**DOI:** 10.3389/fpsyg.2016.00031

**Published:** 2016-01-25

**Authors:** Andrea Valente, Svetlana Pinet, F.-Xavier Alario, Marina Laganaro

**Affiliations:** ^1^Aix-Marseille Université, CNRS, LPC UMR 7290Marseille, France; ^2^Faculty of Psychology and Educational Sciences, University of GenevaGeneva, Switzerland

**Keywords:** ERPs, topographic analysis, word production, reading aloud, object naming

## Abstract

Differences between the cognitive processes involved in word reading and picture naming are well established (e.g., visual or lexico-semantic stages). Still, it is commonly thought that retrieval of phonological forms is shared across tasks. We report a test of this second hypothesis based on the time course of electroencephalographic (EEG) neural activity, reasoning that similar EEG patterns might index similar processing stages. Seventeen participants named objects and read aloud the corresponding words while their behavior and EEG activity were recorded. The latter was analyzed from stimulus onset onward (stimulus-locked analysis) and from response onset backward (response-locked analysis), using non-parametric statistics and the spatio-temporal segmentation of ERPs. Behavioral results confirmed that reading entails shorter latencies than naming. The analysis of EEG activity within the stimulus-to-response period allowed to distinguish three phases, broadly successive. Early on, we observed identical distribution of electric field potentials (i.e., topographies) albeit with large amplitude divergences between tasks. Then, we observed sustained cross-task differences in topographies accompanied by extended amplitude differences. Finally, the two tasks again revealed the same topographies, with significant cross-task delays in their onsets and offsets, and still significant amplitude differences. In the response-locked ERPs, the common topography displayed an offset closer to response articulation in word reading compared with picture naming, that is the transition between the offset of this shared map and the onset of articulation was significantly faster in word reading. The results suggest that the degree of cross-task similarity varies across time. The first phase suggests similar visual processes of variable intensity and time course across tasks, while the second phase suggests marked differences. Finally, similarities and differences within the third phase are compatible with a shared processing stage (likely phonological processes) with different temporal properties (onset/offset) across tasks. Overall, our results provide an overview of when, between stimulus and response, word reading and picture naming are subtended by shared- versus task-specific neural signatures. This in turn is suggestive of when the two tasks involve similar vs. different cognitive processes.

## Introduction

It is well known that the time required to name an object is greater than the time required to read aloud its name (Cattell, 1885). This effect can resist even intensive training (Fraisse, 1964; Theios and Amrhein, 1989; Ferrand, 1999). This appears to be one of the clearest and most ubiquitously replicated pieces of evidence in psycholinguistics, and it has been object of scientific investigation since the very early stages of psycholinguistics.

Given that no controversy exists on such observation, efforts have been conveyed toward the understanding of its causes. Some accounts relied on differences at the level of the visual discriminability of the stimuli (see for instance Theios and Amrhein, 1989). Words are more easily processed perceptually if compared to a pictorial representation of the object they refer to. Nonetheless, several studies demonstrated that words and pictures are equally discriminable stimuli, and therefore discriminability cannot really account for differences in response speed (e.g., Fraisse, 1984; Theios and Amrhein, 1989).

Alternatively, it has been argued that pictures can be named in different ways, while only one response is possible for a written word (Fraisse, 1969; Theios, 1975), the so called “uncertainty factor” (see Ferrand, 1999).

If we narrow the discussion down to specific processing accounts, it has been submitted that naming a picture differs from word reading in some fundamental cognitive aspects. For instance, naming a depicted object would require some additional processing steps reading a word does not call for. When presented with a picture, the speaker has to recognize the object it represents. This is achieved by retrieving its visuo-semantic properties, prior to selection of the corresponding lexical item (e.g., Glaser, 1992). Conversely, reading a word aloud (in the alphabetic languages most commonly studied), can in principle be done by performing a conversion of the graphemes (the written form of a phoneme) in a phonological output, dispensing with the need of an extensive retrieval of semantic information necessary for object recognition (Coltheart et al., 1993; Indefrey and Levelt, 2004; Price et al., 2006).

Evidence has also been reported of an early activation of semantic information on presentation of both auditory (e.g., Pulvermüller et al., 2005) and written (Dell’Acqua et al., 2007) words and non-words. Furthermore, a thorough retrieval of semantic information is expected in reading aloud tasks involving semantic categorization and decision (see for instance Mulatti et al., 2009). Nonetheless, the involvement of semantics should occur with different weights in picture naming vs. word reading tasks in which participants are instructed to read aloud words appearing on a screen, and this has been repeatedly used as one of the arguments to explain, why naming a picture takes longer than reading the corresponding word (Theios and Amrhein, 1989, but see Janssen et al., 2011).

Despite these differences, some processing steps do appear to be equally needed for successful performance in both picture naming and word reading. For instance, it is commonly thought that phonological processing – that is, the retrieval of the word’s phonological form necessary to implement the articulatory gestures – is shared in both tasks (e.g., Coltheart et al., 2001). In accordance to this, picture naming and word reading are assumed to involve similar outputs triggered by distinct inputs. Both behavioral and neuroimaging data have been marshaled in support of this hypothesis. Roelofs (2004) investigated initial segment planning – measured as the facilitation in reaction times – by using mixed vs. blocked picture naming and word reading trials. The idea behind the paradigm was that if the phonological processing stage were common to both picture naming and word reading, then a phonological facilitation should be observed not only when the tasks are blocked, but also when they are mixed (i.e., pictures and words alternated). Results supported the authors’ hypothesis. Price et al. (2006) investigated the neuronal basis of object naming compared with word reading in a functional neuroimaging (fMRI) study. Results revealed that the areas of speech production selectively activated during object naming were the same that were recruited during the reading of words, though in word reading the activation was comparatively enhanced.

The converging anatomical substrate supports the assumption that retrieval of the phonological form of the word to be uttered is comparable, whether one has to name an object or to read the corresponding word. In this context, the primary aim of the present study is to characterize the contrastive temporal signatures of picture naming and word reading; we do so by comparing directly the electroencephalographic (EEG) correlates of the two tasks, while taking into account their typically different response latencies. These contrasts can ultimately clarify the contrasts between shared and specific processes underlying the two tasks.

As noted above, picture naming and word reading differ on at least three major aspects: the specific cognitive processes they are assumed to involve, the moment in time in which such processes are triggered, and the speed at which responses are given. For this reason, ERP waveform analysis will be associated with topographic pattern (microstate) analysis (Murray et al., 2008). Topographic analysis is a reference-free methodology useful to partition the evoked potentials in periods of stable topographic activity, corresponding to “a period of coherent synchronized activation of a large-scale neuronal network,” which represent “the basic building blocks of information processing” (Brunet et al., 2011). It is useful to clarify that highlighting a serial succession of periods of stable topographic activity does not constitute an endorsement of a serial organization of the processing stages envisaged by some cognitive models. Each period of topographic stability can surely conglomerate the brain’s parallel processing of different types of information. Still, it is thought to represent a functional integrative step necessary to accomplish the cognitive task at hand (Brunet et al., 2011). This point has a particular relevance given the issues addressed in the present study; topographic analysis can inform us on whether specific topographic maps are present both in picture naming and in word reading, with additional information on their temporal signatures, which is useful to draw conclusions on cross-task differences in stages of information processing.

According to the theoretical accounts concerning the cognitive processes underlying picture naming and word reading, cross-task ERP differences should be detectable in early time windows following visual encoding, since this specific time window is thought to involve cross-task specificities (extensive lexico-semantic vs. primarily ortho-phonological processing). In previous ERP studies, evidence has been reported that these task-specific processing stages are engaged in the time window following visual encoding and preceding retrieval of the phonological codes (Bentin et al., 1999; Simon et al., 2004; Indefrey, 2011; Hauk et al., 2012). In contrast, between-task similarities in the electrophysiological signatures are expected in later time windows approaching response articulation, in which it is likely to expect a primary involvement of phonological codes in both tasks.

## Materials and Methods

### Method

The present study was approved by the local ethics committee of the faculty of Psychology and Educational Sciences of the University of Geneva, and carried out in accordance with the recommendations of the Swiss Federal Act on Research involving Human Beings. All participants gave their written informed consent in accordance with the Declaration of Helsinki.

A total of 24 healthy participants recruited among university students (aged between 18–30, mean: 22,8, *SD*: 3,5; three men) participated in the study. The scoring and data analysis led to the exclusion of seven participants, while 17 were retained (see below for details).

All participants gave informed consent and received monetary compensation for their participation in the study. They were right-handed as assessed by the Edinburgh Handedness Scale (Oldfield, 1971). They were all native French speakers.

### Material

We used a set of 120 black-and-white line drawings and their corresponding words extracted from two French databases (Alario and Ferrand, 1999; Bonin et al., 2003).

All pictures had a name agreement above 75% (mean = 92.5%). This was done to minimize the odds of atypical responses. The stimuli were monosyllabic (*N* = 40), bisyllabic (*N* = 60), and trisyllabic (*N* = 20) words, of lexical frequency ranging from 0.13 to 227 occurrences per million words (mean = 17.3) according to the French database Lexique (New et al., 2004). The same 120 items (i.e., words and pictures) were presented in each task. Pictures consisted of 280 × 280 pixels black-line drawings, while the corresponding words were displayed in Courier New 18-point font, in white color on a gray background.

### Procedure

Participants were tested individually in a soundproof cabin. They sat at about 60 cm from the computer screen. The stimuli were presented with the E-Prime software (Psychology Software Tools, Inc., [E-Studio], 2012) and appeared in a pseudo-randomized order, that is semantic and phonological neighbors were never presented in strict succession.

All participants were familiarized with the pictures before performing the task. They were shown the set of pictures associated with the corresponding written words in order to resolve any doubts or non-recognitions. To familiarize participants with the task, a training part was administered before the experimental session involving trials with the same temporal sequence than those used in the experiment. The order of picture naming and word reading blocks was counterbalanced across participants.

#### Picture Naming

Each trial started with a fixation cross presented for 500 ms, followed by a 200 ms blank screen and finally by the picture which was displayed for 1500 ms on a gray background.

Participants were instructed to name the pictures overtly, as quickly and accurately as possible while responses were recorded with a microphone.

The maximum delay conceded for articulation was 2000 ms. Responses not given within this time interval were classified as “no responses.”

#### Word Reading

The timing sequence of the trials was identical to the picture naming task with words, instead of the pictures, presented on the screen for 1000 ms. This shorter duration was chosen, because faster response latencies were expected in word reading.

#### Processing of Verbal Responses

Behavioral analyses were conducted on the sample of participants retained for the ERP analysis, after exclusion of participants with artifact-contaminated EEG signal. Seventeen participants (aged 18–29, mean: 22.6, *SD*: 3,1) were finally retained.

Response latencies, defined as the time elapsing between stimuli (picture and word) onset and the acoustic onset of response articulation, were estimated with Check Vocal (Protopapas, 2007). This software allows to visualize, both speech waveforms and spectrograms of each response in order to identify the speech onset.

#### EEG Acquisition and Processing

We used the Active-Two Biosemi EEG system (Biosemi V.O.F., Amsterdam, Netherlands) in its high-density montage, with 128 channels covering the scalp. Signals were sampled at 512 Hz with a band-pass filtering set between 0.16 and 100 Hz.

The EEG signal was calculated against the average reference and bandpass-filtered to 0.1–40 Hz.

Each trial in both tasks and for each participant was inspected visually for various forms of artifact contamination (blinks, eye movements, skin, or muscular artifacts) and noisy channels. An automated selection criterion, highlighting channels displaying amplitudes oscillations reaching ±100 μV, was also applied. Trials containing artifacts were excluded from ERP averaging. As a heuristic criterion, only participants with at least 60 usable trials in each task were retained for further analyses. For the waveform analysis (detailed below), stimulus-aligned epochs were extracted with a baseline correction of 100 ms; for the topographic analysis, no baseline correction was applied to the ERPs.

In both picture naming and word reading, stimulus-aligned and response-aligned epochs of 400 ms were averaged across participants in both conditions in order to obtain a Grand-Mean of ERPs for each task. Stimulus-aligned epochs were locked to picture onset in the picture naming task and to word onset in the word reading task. Response-aligned epochs were locked to the onset of articulation in both tasks.

#### EEG Analyses

Electroencephalographic analyses were performed in two main steps: waveform analysis and topographic pattern analysis.

##### Waveform analysis

First, a sample-wise ERP waveform analysis was performed on both stimulus- and response-locked ERPs in order to assess at which time points significant amplitude differences were present between picture naming and word reading. We compared both conditions time-locked to the stimulus onward (from –100 to 400 ms) and to vocal onset backward (up to –400 ms).

ERP waveforms were analyzed by means of a cluster-based non-parametric analysis (Maris and Oostenveld, 2007). This technique allows to compare each point in time (∼every 2 ms) and channel between two conditions while correcting for multiple comparisons by taking into account spatial (four neighboring channels) and temporal (two successive time-points) adjacency: only clusters over a given significance level were kept. The level of significance was determined by building a distribution stemming from the data itself by successive random permutations of the two experimental conditions (picture naming and word reading).

##### Topographic pattern analysis

Amplitude differences between experimental conditions within a given time-window may have different causes. This is because the clustering algorithm used to partition the ERP data only considers the spatial configuration of activity (relative intensity across electrode sites), but not the absolute intensity at each electrode site. Significant differences may be detected when the same scalp electric fields are present in overlapping time intervals but with different intensity. Amplitude differences can also occur when spatial differences in the distribution of the potentials at the scalp are present (different topographic maps, revealing different brain generators), or even if the same scalp electric fields are present, but appear in different time-windows between conditions (i.e., if they are shifted in time). To assess the precise origin of the amplitude differences determined above, we performed two analyses.

First, we ran a sample-wise topographic analysis of variance (TANOVA). This method identifies the time periods during which topographic differences were present between tasks. The TANOVA is a non-parametric randomization test of the global dissimilarity measures between different experimental conditions or groups (Murray et al., 2008), useful to determine at which time points between stimulus and response different scalp topographies are present across the conditions of interest. As an empirical criterion, only topographic differences lasting more than 30 ms were considered and an alpha value of 0.01 was adopted.

Secondly, a microstate analysis (spatio-temporal segmentation) was performed on the ERP Grand-Means to identify the electrophysiological and temporal signatures of the building blocks of information processing present in picture naming and in word reading. This methodology clusters the ERP Grand-Means in a series of periods of quasi-stable electrophysiological activity (template maps) that best explain the global variability of the dataset. Only the spatial configuration of the maps, but not their intensity, is taken into account. Additional information can be obtained concerning the duration and other dependent measures of the stable periods in different conditions or groups. Any modification of the spatial configuration of the electric field measured at the scalp is unequivocally interpreted as indicating a different pattern of cerebral sources, namely a difference in the information processing the brain is engaged in (e.g., Pascual-Marqui et al., 1995). The microstate analysis was performed as follows. A spatio-temporal segmentation was computed on the Grand-Mean ERPs of both picture naming and word reading, in both the stimulus- and response-aligned conditions separately, using an optimized agglomerative clustering algorithm, the topographic atomize and agglomerate hierarchical clustering algorithm (TAAHC: Murray et al., 2008).

In both the response- and stimulus-aligned conditions, the ERP Grand-Means of both picture naming and word reading were subjected to the clustering together, i.e., template maps were computed from a concatenation of the Grand-Means of both tasks. This was done with the purpose of maximizing information about similarities and differences in the ERP signal.

The spatio-temporal segmentation partitions the ERP Grand-Means in a series of periods of quasi stable electrophysiological activity, which summarize the data and are useful to determine which template map best explains the participants’ ERPs in each experimental condition. A temporal post-processing was also performed, allowing to reassign segments with a short duration (less than 30 ms) to neighboring clusters and to merge together very highly spatially correlated maps (above 0.92).

At the end of each segmentation, we are provided with a set of quality measures indicating, which is the best segmentation among alternatives. Cross-validation and Krzanovski–Lai criterion were used to this end. Cross-validation is the ratio between global explained variance (GEV) and degrees of freedom (number of electrodes). Since this measure gets less reliable as the number of electrodes increases, it is associated with the Krzanovski–Lai criterion, which computes the dispersion of the segmentation (see Murray et al., 2008). Segmentations corresponding to both the CV minimum and Krzanovski–Lai measure peak are usually the most reliable, as they represent a reasonable compromise between compression of the data and high GEV.

Once the group-averaged ERPs is segmented into a series of template maps, these can be tested by *back-fitting* them in the individual subject-averaged ERPs. This *back-fitting* procedure assigns each time point of each of the individual subjects ERPs to the Grand-Average’s template maps it best correlates with. This yields a measure of the template maps’ presence in each condition and allows to establish how well a cluster map explains individual patterns of activity (GEV). Moreover, it provides information on map duration, first onset and last offset, Global Field Power and other dependent measures, which can subsequently be used for statistics.

These analyses were performed with the Cartool software (Brunet et al., 2011).

## Results

### Behavioral Results

In both tasks, atypical responses (i.e., errors) and non-responses were excluded from further analysis (1,3% of the data). Response latencies above and below 3 SD were calculated for each participant in each task and excluded from further analysis (2% of the data).

On average, participants named pictures slower (mean RTs = 872 ms, *SD* = 205 ms) than they read the corresponding words (mean RTs = 560 ms, *SD* = 101 ms). The 312 ms difference was significant [*t*(16) = 18,799, *p* < 0.001].

### ERP analysis

#### Stimulus-Aligned

**FIGURE 1:**
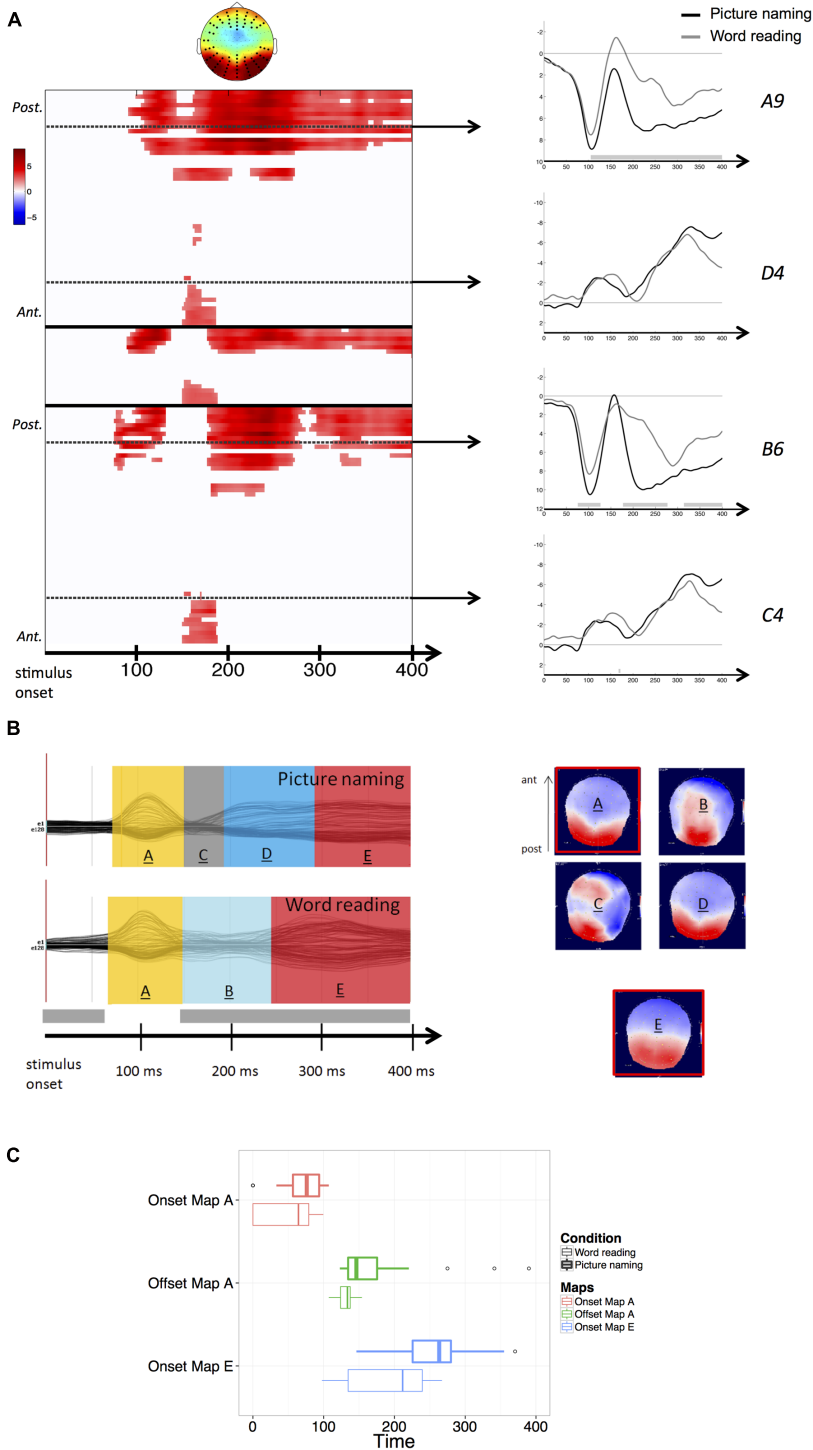
**(A)** Results of the stimulus-aligned waveform analysis. Values are masked by results of cluster-based non-parametric analysis: only significant values are plotted. Within the left panel, upper part corresponds to left hemisphere electrodes, middle part to midline electrodes, and lower part to right hemisphere electrodes; within each part, electrodes are ordered from posterior to anterior. Dashed lines outline representative electrodes, which time course is plotted separately (picture naming in black, word reading in gray). The topography represents the spatial distribution of the effect over each cluster (black dots outline electrodes within each cluster). **(B)** Results of the spatio-temporal segmentation on the stimulus-locked ERP Grand-Means of both tasks. Each period of topographic stability is displayed in the color bars with the information about its time course. The corresponding topographies are listed on the right (positive values in red, negative values in blue), with the common topographies marked in red. The gray bar on the temporal axis represents the periods of topographic difference between tasks, as revealed by the TANOVA. **(C)** Boxplots of distributions of individual onsets of maps A and E and offsets of map A, extracted from the back-fitting procedure for both picture naming (bold lines) and word reading (thin lines). Zero of times represent stimulus presentation.

In the stimulus-aligned condition (from 0 to 400 ms after stimulus onset) significant differences in amplitudes (*p* < 0.05) were observed between word reading and picture naming throughout the whole time-window of processing. These differences were particularly present over posterior electrodes and bilaterally from 100 to 400 ms post-stimulus (**Figure 1A**).

Results of the TANOVA showed that topographic differences between tasks also stretched across the whole time-window of processing, with the exception of the period comprised between about 75 and 150 ms after stimulus onset (see **Figure 1B**), corresponding to the temporal signature of the P1 component map.

The spatio-temporal segmentation of the stimulus-aligned Grand-Means explained 95,81% of the Global Variance, and revealed the presence of a total of six template maps. In **Figure 1B**, the five template maps starting from the P1 component map onward (map labeled “A”) are shown. In picture naming, the topographic configurations present in the P1 range (map “A”) and later in the 200–300 ms time window (map “D”) were highly correlated spatially and therefore labeled with the same template map by the clustering algorithm. When the same template map appears repeatedly in different non-overlapping time windows of the same Grand-Mean, it does not reflect comparable neuronal activity (e.g., Michel et al., 2009). For this reason, the later map has been relabeled differently in the figure, as it likely reflects a qualitatively different step of information processing following early visual encoding.

The application of the clustering algorithm resulted in a sequence of topographic maps, depicted in **Figure 1B** for the grand-averages of each task. Results of the spatio-temporal segmentation revealed that in an early time-window (comprised between about 75 and 150 ms after stimulus onset and thus compatible with visual encoding), the same topographic map (labeled “A”) was present in the grand-averages of both tasks. In the waveform analysis, higher amplitudes were detected in word reading compared with picture naming (**Figure 1A**). The TANOVA corroborated the results of the spatio-temporal segmentation, revealing that the same topographic maps were predominant across tasks in the considered time-window (75–150 ms). A back-fitting was performed in the time window comprised between 0 and 400 ms from stimulus onset to test for the onsets, offsets and durations of map “A” across participants in both tasks. Results revealed that map “A” had a slightly later onset in picture naming (mean onset: 66 ms after picture onset) with respect to word reading (mean onset: 50 ms after word onset). A Wilcoxon signed-rank test proved the difference to be marginal (*z* = –1,818, *p* = 0.07). Map “A” also displayed a later offset in picture naming (mean offset: 155 ms after picture presentation) compared with word reading (mean offset: 132 ms after word presentation). The difference proved to be significant (*z* = –2,301, *p* < 0.05). Finally, no differences were found in map duration across tasks (*z* = –1,086, *p* = 0.278). **Figure 1C** illustrates the distributions of the individual onsets and offsets of map “A” in both picture naming and word reading.

The time window following visual encoding (starting from about 150 ms onward) was characterized by extensive amplitude differences, mainly located on posterior sites. In this time window, substantial topographic cross-task differences were detected. A back-fitting performed on the time window comprised between 160 and 300 ms after stimulus onset revealed that map “D,” characterized by posterior positivity and anterior negativity (**Figure 1B**), was significantly more present in picture naming compared with word reading (Pearson Chi Square computed on map presence across individuals: χ^2^ = 14.43, *p* < 0.001). In picture naming, Map “D” explained the 10% of the variance in the considered time-window (160–300 ms). The posterior characterization of amplitude differences as revealed by the waveform analysis seems consistent with the fact that in picture naming, map “D” was predominant in the considered time-window. Conversely, map named “B” was significantly more present in the word reading task (χ^2^ = 6,10, *p* < 0.05) and explained only the 3% of the variance in the time window comprised between 160 and 300 ms after word onset. The low explained variance can be attributed to the rapidly changing spatial configuration of map “B,” which is likely to be due to the unstable and transitory nature of the ERP activity in the considered time-window.

The back-fitting revealed that map named “C” had a negligible presence in individual ERPs. This is probably due to the transitional and unstable nature of this topographic map (see **Figure 1B**).

Amplitude differences were then sustained in the time window from about 250 ms to the end of the stimulus-locked analysis, corroborated by topographic differences identified by the TANOVA. These differences are however likely to be due to the very different time course of the processing stages specific of each task. In fact, the spatio-temporal segmentation performed on this time window revealed the presence, in both tasks, of the same period of topographic stability (map labeled “E”) characterized by posterior positivity and anterior negativity. This common map displayed noticeably different time courses between tasks. The back-fitting performed on the time window comprised between 100 and 400 ms after stimulus onset revealed that map “E” (explaining the 22% of the variance across tasks in the considered time-window) displayed an earlier onset in word reading (mean onset: 187 ms after word presentation) with respect to picture naming (mean onset: 252 ms after picture presentation). **Figure 1C** illustrates the distribution of the individual onsets of the common map “E” between tasks. A Wilcoxon signed-rank test proved the cross-task difference in the onset to be significant across participants (*z* = –2,342, *p* < 0.05).

#### Response-Aligned

**FIGURE 2:**
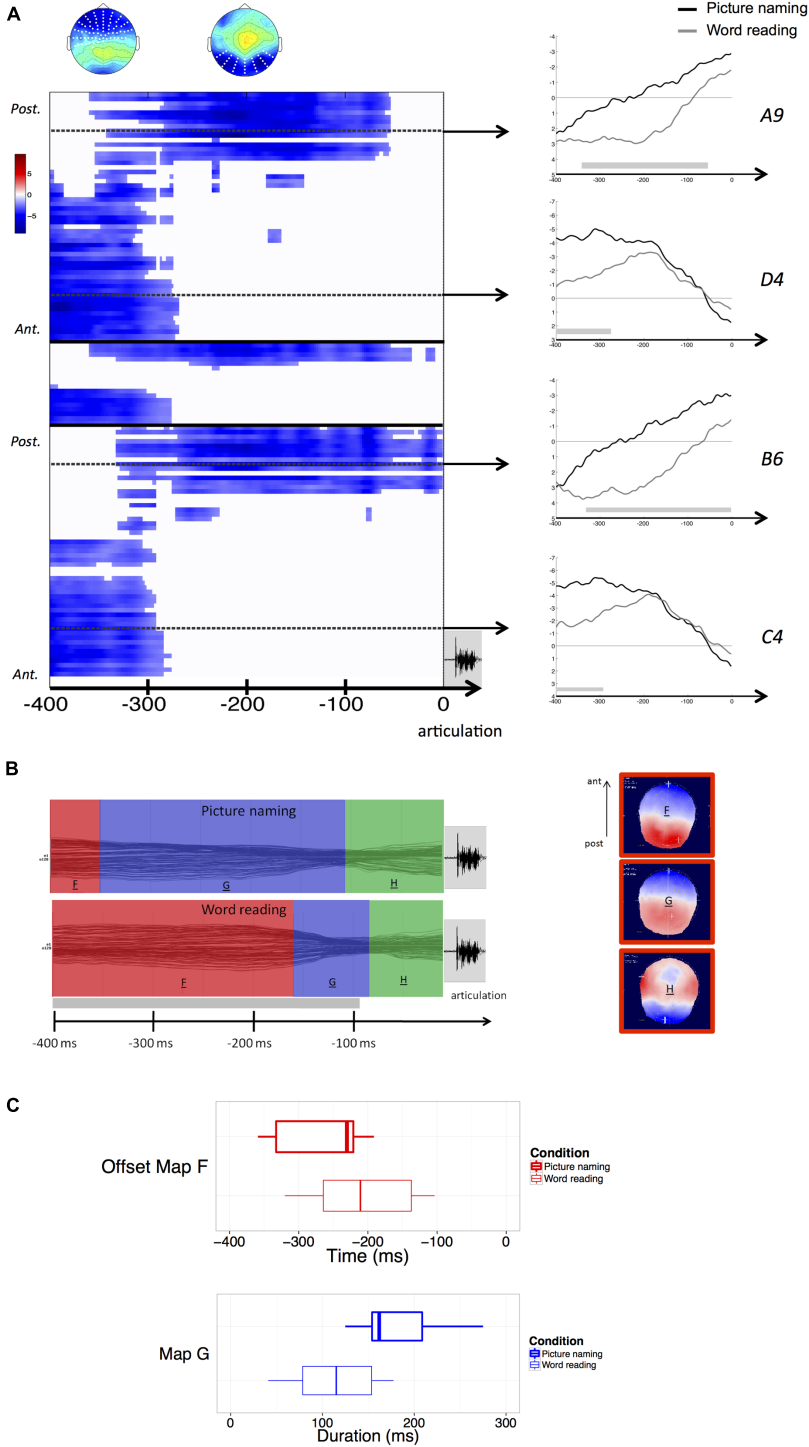
**(A)** Results of the response-aligned waveform analysis. Values are masked by results of cluster-based non-parametric analysis: only significant values are plotted. Within the left panel, upper part corresponds to left hemisphere electrodes, middle part to midline electrodes, and lower part to right hemisphere electrodes; within each part, electrodes are ordered from posterior to anterior. Dashed lines outline representative electrodes, which time course is plotted separately (picture naming in black, word reading in gray). The topography represents the spatial distribution of the effect over each cluster (white dots outline electrodes within each cluster). **(B)** Results of the spatio-temporal segmentation on the response-locked ERP Grand-Means of both tasks. Each period of topographic stability is displayed in the color bars with the information about its time course. The corresponding topographies are listed on the right (positive values in red, negative values in blue), with the common topographies marked in red. The gray bar on the temporal axis represents the periods of topographic difference between tasks, as revealed by the TANOVA. **(C)** Boxplots of distributions of individual offsets of maps F and durations of map G, extracted from the back-fitting procedure for both picture naming (bold lines) and word reading (thin lines). Zero of times in the boxplot of the offset of map F represents voice onset.

In the response-aligned condition (from –400 ms to the vocal response onset), significant amplitude differences (*p* < 0.05) were observed between word reading and picture naming throughout the whole time-window of interest. Differences were observed earlier over anterior electrodes (from –400 to –300 ms) and more posteriorly in the following time-window closer to articulation. Again, effects were bilateral (**Figure 2A**).

The TANOVA revealed an extended period of topographic difference, stretching across the whole time-window of processing with the exception of the last period starting about 100 ms prior to the onset of articulation.

The spatio-temporal segmentation revealed the presence of three template maps (**Figure 2B**) – labeled “F,” “G,” and “H” – explaining 94,5% of the Global variance.

The template map labeled “F” corresponds to the common map (“E”) in the stimulus-aligned condition. These maps were, in fact, spatially correlated above 0.99.

All the three maps were common to both tasks, but maps “F” and “G” displayed different time courses. A back-fitting procedure was carried out in the time-window comprised between –400 and –60 ms before response articulation, revealing that in word reading the map labeled “F,” explaining the 20% of the variance in the considered time-window, displayed an offset much closer to response articulation (mean map offset: 184 ms before articulation) compared with picture naming (mean map offset: 257 ms before articulation). This result proved to be significant across participants (*z* = –2,580, *p* = 0.01). A second back-fitting was performed in the time-window comprised between –380 and –60 ms to test for the duration of map “G” across tasks. The results revealed that map “G” had a longer duration in picture naming (mean duration: 243 ms) compared with word reading (mean duration: 113 ms). The result was significant (*z* = –3,297, *p* < 0.01).

**Figure 2C** illustrates the distribution of the offsets of map “F” and the duration of map “G” across participants and for each task. It is worthy of notice that the mean maps offset and duration calculated across participants might be different when compared to the mean onsets of the same maps in the ERP Grand-Means, because of variability across participants.

## Discussion

Participants named pictures slower than they read the corresponding words. This result is consistent with a vast literature (e.g., Cattell, 1885; Fraisse, 1964, 1969; Theios and Amrhein, 1989; Ferrand, 1999; Price et al., 2006; Riès et al., 2012).

Results of the ERP analysis will be discussed by focusing on three successive phases, tentatively defined on the basis of the degree of cross-task similarities and differences in the observed EEG patterns. These phases correspond roughly to the time-window between 75 and 150 ms after stimulus onset, a post-visual time-window ranging from about 150 to 250 ms, and a later time-window encompassing ERP activity close to response articulation.

No cross-task differences were detected in terms of the spatial configurations at the scalp present in the time-window between 75 and 150 ms after stimulus onset. Nevertheless, the visual topographies displayed a slightly different time course across tasks, and the waveform analysis showed that the visual ERP component in word reading displayed higher amplitude with respect to picture naming. These observations possibly stem from the different recruitment of the visual cortex due to the different types of visual stimuli (pictures vs. words), during a period in which visual encoding of the stimuli was presumably predominant. The observation of such cross-task differences in intensity and time course of the early map “A” does not stand in contrast with previous evidence that visual processing can occur in parallel with the cascaded activation of other pertinent informational codes. In this respect, evidence has been reported of an early activation of semantic information in both visual objects (e.g., Miozzo et al., 2015) and written words (e.g., Pulvermüller et al., 2005; Dell’Acqua et al., 2007). In accordance with this hypothesis, the shared “visual” map might possibly encompass a task-dependent variable degree of spreading of activation to semantics.

The time-window immediately following visual encoding (from about 150 to 250 ms) was characterized by extensive cross-task amplitude and topographic differences. In fact, two different scalp topographies were predominantly present between tasks (map “D” in picture and map “B” in word reading in **Figure 1B**). Even though map “B” was characterized by a transitory and rapidly changing spatial configuration, these results point to substantial electrophysiological differences between picture naming and word reading in the “post-visual” time window, which can in turn be interpreted as functional differences in the information processing occurring between tasks. More specifically, naming a picture is thought to require extensive retrieval of the semantic information associated with the stimulus, without which no recognition of the object would be achieved (Glaser and Glaser, 1989; Theios and Amrhein, 1989; Indefrey and Levelt, 2004; Price et al., 2006). The hypothesis that different processing stages are implied between picture naming and word reading has been proposed in a previous ERP study (Yum et al., 2011) in which the authors reported diverging ERP correlates associated with the processing of pictures vs. words in the time-window compatible with the N170 component (between 150 and 200 ms after stimulus presentation).

Amplitude and topographic differences were also detected in the later time-window from about 250 ms from stimulus onset onward. These are mainly due to the remarkable cross-task difference in response latencies and time course of the information processed between tasks. Indeed, the spatio-temporal segmentation on this time window revealed the presence in both tasks of the same period of topographic stability characterized by posterior positivity and anterior negativity. The presence of this shared topographic pattern indicates that the same underlying brain generators were active in both tasks thought with different time courses, namely significantly earlier in word reading with respect to picture naming. The fact that these similar topographic patterns were present in a time-window approaching the onset of response articulation, in which it is likely to expect retrieval of the phonological codes necessary to initiate utterance of the words, gives room to the tentative hypothesis that they, at least partially, convey retrieval of the phonological codes. This interpretation is in line with previous neuroimaging evidence reporting that picture naming and word reading rely on the activation of comparable speech production areas (e.g., Price et al., 2006) and would hence support the hypothesis that the processing of phonology is shared across picture naming and word reading (Roelofs, 2004; Price et al., 2006).

Furthermore, the fact that the shared topographic map displayed an earlier onset (about 70 ms) in word reading compared with picture naming seems to suggest that this specific operation may occur earlier in word reading, which could account for the shorter response latencies observed in this task.

Hypotheses that retrieval of the phonological form occur earlier in word reading with respect to picture naming have been advanced in previous studies by positing a stronger connection between written words and phonological codes (Glaser and Glaser, 1989) and by assuming that contrary to picture naming, in word reading no semantic processing followed by selection of the lexical entry is required (Theios and Amrhein, 1989). In fact, in word reading the phonology could in principle be directly accessed via a grapheme-to-phoneme conversion, whereas in picture naming the retrieval of the phonological form is thought to be conditional to the retrieval of semantic information (e.g., Price et al., 2006) or concomitant to the semantic processing necessary to recognize the depicted object (Miozzo et al., 2015; see also Abdel-Rahman et al., 2003). The earlier onset of the map “E” in word reading would be compatible with theories positing a faster access to phonological codes in word reading with respect to picture naming. Whether this earlier access to phonology in word reading stems from access to phonology from the grapheme to phoneme conversion stage, or to the differential levels of recruitment of semantic information across tasks, the observation might partially account for the shorter latencies observed in word reading compared with picture naming.

It is useful to clarify that more specific interpretations concerning the information processed during the shared periods of stable topographic activity cannot be completely excluded. For instance, one can posit that the activation of phonological codes in word reading might automatically spread to semantics. However, the experimental design here adopted does not allow to extensively test these hypotheses, insofar as no factors capable of affecting phonological or semantic processing were manipulated.

In the response-locked ERPs, amplitude and topographic differences (identified by the TANOVA) are again ascribable to the different time course of the processing stages involved. The results of the segmentation allowed to identify three common periods of topographic stability, two of which displayed very different time courses.

The same topographic map identified in the final stimulus-locked period was detected in the segmentation of the response-locked ERPs. Interestingly, in this case the map displayed an offset much closer to response articulation in word reading compared with picture naming. In other words, the transition between the offset of this topographic map and the onset of articulation was significantly shorter in word reading with respect to picture naming.

Considering that this period of quasi stable electrophysiological scalp activity was common in both tasks, and surmising that it could convey the retrieval of the phonological form of the words, then the transition between this phase and the moment in which articulation could be initiated appears to be faster in word reading compared with picture naming.

This can be explained by intervening pre-articulation monitoring processes specific of picture naming, and could for instance reflect the higher level of uncertainty one has to face when naming a picture compared with reading a word aloud (e.g., Fraisse, 1969; Ferrand, 1999), leading to more cautious responses. For instance in an overt picture naming task, Valente et al. (2014) reported effects of the variables name agreement and image agreement in the time window preceding response articulation, supporting the hypothesis of a monitoring of response before the onset of articulation.

Another possible explanation might be the cascading of information from earlier stages of encoding. Even though the present study was not aimed to tackle directly the issue of cascade processing, one could hypothesize that phonological information can be differentially activated depending on the specific task one has to perform. This could in turn affect the moment in which articulation can be undertaken. Such hypothesis is also consistent with evidence reported by Price et al. (2006) of a higher activation of speech production areas in word reading compared with picture naming. Likewise it has been posited that, in word reading, articulation can be initiated on the basis of more partial information (Hennessey and Kirsner, 1999). Our results are not inconsistent with this assumption, in so far as the transition between the offset of the common topographic map and the onset of articulation was significantly faster when participants had to read words compared with when they had to name pictures.

Although further investigation is required to directly address the issue, this assumption would also reinforce an explanation on why and when word reading is faster than picture naming.

## Conclusion

This study sought to investigate how picture naming and word reading differ over the time course of processing from stimulus to response. We offer evidence that the same periods of stable topographic activity were present across tasks in two time-windows, compatible with processing of visual information and retrieval of the phonological form. The latter period of stable topographic activity, close to response articulation, displayed different time courses across tasks, with an earlier onset with respect to stimulus presentation in word reading than picture naming. This result can be tentatively interpreted as a faster access to phonological codes from written words than pictorial stimuli, which do require an extra semantic stage necessary for object recognition and identification. Likewise, the common topographic map thought to partially convey phonological processing, had an offset closer to response articulation in word reading compared with picture naming, suggesting that response articulation can be initiated comparably faster in word reading, once phonological information becomes available. Altogether, our interpretation provides some indications regarding the temporal origin of faster responses in word reading compared with picture naming.

## Conflict of Interest Statement

The authors declare that the research was conducted in the absence of any commercial or financial relationships that could be construed as a potential conflict of interest.
